# m^6^A mRNA Methylation Was Associated With Gene Expression and Lipid Metabolism in Liver of Broilers Under Lipopolysaccharide Stimulation

**DOI:** 10.3389/fgene.2022.818357

**Published:** 2022-02-25

**Authors:** Feng Guo, Yanhong Zhang, Jinyou Ma, Yan Yu, Qiuxia Wang, Pei Gao, Li Wang, Zhiyong Xu, Xiaobing Wei, Mengna Jing

**Affiliations:** ^1^ College of Animal Science and Veterinary Medicine, Henan Institute of Science and Technology, Xinxiang, China; ^2^ Postdoctoral Research and Development Base, Henan Institute of Science and Technology, Xinxiang, China

**Keywords:** m^6^A-sequencing, chicken, liver, inflammation, lipid metabolism, m^6^A-sequencing

## Abstract

Hepatic inflammation is always accompanied with abnormal lipid metabolism. Whether N^6^-methyladenosine (m^6^A) mRNA methylation affects irregular inflammatory lipid level is unclear. Here, the m^6^A modification patterns in chicken liver at the acute stage of LPS-stimulated inflammation and at the normal state were explored *via* m^6^A and RNA sequencing and bioinformatics analysis. A total of 7,815 m^6^A peaks distributed in 5,066 genes were identified in the normal chicken liver and were mostly located in the CDS, 3′UTR region, and around the stop codon. At 2 h after the LPS intraperitoneal injection, the m^6^A modification pattern changed and showed 1,200 different m^6^A peaks. The hyper- and hypo-m^6^A peaks were differentially located, with the former mostly located in the CDS region and the latter in the 3′UTR and in the region near the stop codon. The hyper- or hypo-methylated genes were enriched in different GO ontology and pathways. Co-analysis revealed a significantly positive relationship between the fold change of m^6^A methylation level and the relative fold change of mRNA expression. Moreover, computational prediction of protein–protein interaction (PPI) showed that genes with altered m^6^A methylation and mRNA expression levels were clustered in processes involved in lipid metabolism, immune response, DNA replication, and protein ubiquitination. CD18 and SREBP-1 were the two hub genes clustered in the immune process and lipid metabolism, respectively. Hub gene AGPAT2 was suggested to link the immune response and lipid metabolism clusters in the PPI network. This study presented the first m^6^A map of broiler chicken liver at the acute stage of LPS induced inflammation. The findings may shed lights on the possible mechanisms of m^6^A-mediated lipid metabolism disorder in inflammation.

## Introduction

N^6^-methyladenosine (m^6^A) is the most common RNA modification in mammalian mRNA. With MeRIP-Seq technology, more than 7,000 mammalian genes were detected with m^6^A modification (Meyer et al., 2012). These m^6^A sites preferentially distribute near the stop codons and in the 3′UTRs and are highly conserved among humans, mice, and pigs (Dominissini et al., 2012; Wang et al., 2018). m^6^A methylation is reversible, and the methyltransferase complex (writers) comprises the enzymatic core components METTL3 and METTL14 and many co-factors such as WATP and VIRMA. Two demethylases called erasers, FTO and ALKBH5, can remove the methyl group. Some proteins, called m^6^A readers, can recognize this epimark and then participate in the downstream regulatory processes. Among the m^6^A readers, the YTH-domain family 1 (YTHDF1), YTHDF2, and YTHDF3 proteins can directly bind to the m^6^A position to control the translation and decay of their target mRNA in cytosol. YTHDF1-3 are the three YT521B (YTH) family of proteins, YTHDF1 increases the translational rates of its mRNA targets (Wang et al., 2014), YTHDF2 induces mRNA decay (Du et al., 2016), and YTHDF3 promotes the function of YTHDF1 and YTHDF2 (Shi et al., 2017).

m^6^A function has been widely studied. *In vitro* depletions of the modulators of m^6^A have unfolded the function of this epigenetic marker in many biological processes, including the inflammation and lipid metabolism. LPS stimulation can induce the expression of METTL3 and m^6^A level in human dental pulp cells. The alternative splicing of Myd88 upon LPS stimulation is also regulated by METTL3, which may play an important role in the regulation of inflammation (Feng et al., 2018). YTHDF2 regulates the LPS-induced cytokines expression in macrophages, with MAP2K4 and MAP4K4 as the target gene (Yu et al., 2019). DsDNA- or HCMV-triggered IFNB expression is controlled by m^6^A modulators, METTL14 and ALKBH5. METTL14 depletion upregulates IFNB mRNA and reduces virus reproduction (Rubio et al., 2018).

m^6^A also regulates lipid metabolism. METTL3–METTL14–WTAP methyltransferase complex positively regulates the adipogenesis in 3T3-L1 cells by controlling the cell cycle (Kobayashi et al., 2018). Silencing FTO in preadipocytes increases the m^6^A levels of cell-trolling genes. The reader, YTHDF2, reads the increased m^6^A modulation and decays the mRNAs, leading to failed adipogenesis (Wu et al., 2018). METTL3 knockdown in murine liver cells decreases PPARα m^6^A abundance but increases PPARα mRNA lifetime and expression, thus reducing lipid accumulation in cells *in vitro*. Mechanistically, YTHDF2 binds to PPARα to mediate its mRNA stability to regulate lipid metabolism (Zhong et al., 2018). These studies showed the deep involvement of m^6^A modulators in lipid metabolism regulation.

Lipid homeostasis is disrupted during systemic inflammation. However, the mechanism of inflammatory factor–induced hepatic lipid metabolism disorder remains unclear. Our previous study reported that the LPS-induced inflammation in broiler liver was accompanied by higher FTO protein level and lower CPT1 m^6^A methylation level (Zhang et al., 2016). Lu et al. in 2018 found that LPS treatment increases the total m^6^A level in piglet livers and affects the gene expression of its modulators (Lu et al., 2018). They also found the aberrant expression of lipid related genes. Although the m^6^A level of lipid-associated genes was not examine, m^6^A possibly plays an important role in inflammation-induced aberrant hepatic lipid metabolism because of its function in adipogenesis and innate immunity. In the present work, the global m^6^A profiles of broiler liver was analyzed at normal and inflammation state to determine the possible mechanisms of m^6^A-mediated lipid metabolism disorder in inflammation.

Chickens produce 90% lipid in the liver (Leveille et al., 1975) and are more often to be exposed to environmental inflammation stimuli and threatened by the inflammation-induced abnormality in lipid metabolism. In this study, MRIP-Seq was used to present the chicken m^6^A modification landscape in the mRNA after LPS stimulation. Highly diverse m^6^A modification patterns between these two groups were found. The abnormal m^6^A modifications in LPS-treated samples were proved to be associated with the gene expression of genes and related pathways related to inflammation and lipid metabolism. This study serves as a guide to further investigate the potential roles of m^6^A modification in inflammation-induced abnormal lipid metabolism.

## Materials and Methods

### Animals and Experimental Design

All procedures in this study were carried out in accordance with the Chinese Guidelines for Animal Welfare and Experimental Protocol and were approved by the Institutional Animal Care and Use Committee of Henan Institute of Science and Technology. Sixty fertilized Ross eggs were incubated in an automatic incubator with a temperature of 37°C ± 0.5°C and a relative humidity of 65%. Two weeks after hatching, chicks were randomly divided into Control and LPS group. Chicks in the LPS group were injected intraperitoneally with LPS from *Escherichia coli* 055:B5 (L4524, Sigma-Aldrich) at a dose of 1 mg/kg body weight. Chicks in the Control group were injected with the same volume of PBS. Two and 24 h after the treatment, blood samples were obtained and the plasma were separated, and livers were obtained and frozen in liquid nitrogen and stored at −80°C. Triglycerides and cholesterol content in plasma and liver were measured using the GPO-PAP method.

### Total RNA Extraction and Real-Time PCR

Total RNA was isolated by using TRIzol reagent (15596026, Invitrogen) according to the manufacturer’s introduction. By treating with DNase I (D2215, Takara), the potential genomic DNA contamination was eliminated. Two micrograms of DNase I treated RNA was used to obtained cDNA by using the GoScript Reverse Transcription System (A5001, Promega). cDNA was diluted (1:15) and proceed to real-time PCR analysis, and the TB Green *Premix Ex Ta* Ⅱ (RR820A, TaRaKa) was used for PCR amplification. The following standard PCR conditions were carried out for all transcripts: 95°C for 2 min; 35 cycles of 95°C for 15 s, 60°C for 30 s, and 72°C for 30 s; one cycle of 95°C for 15 s, 60°C for 15 s, and 95°C for 15 s. The last cycle was carried out to provide the melt curve for assessment of the specificity of amplification. Primers used in this study were listed in Supplementary Table S1, and chicken β-actin was selected as a reference gene. The method of 2^−ΔΔCt^ was used to analyze the data. Sequences of the primers used in real-time PCR were listed in Supplementary Table S1.

### Global m^6^A Quantification

Two hundred micrograms of total RNA from the liver of both the Control and LPS groups were subjected to m^6^A quantification using the EpiQuik m^6^A RNA Methylation Quantification Kit (Colorimetric) (Epigentek, P-9005) following the manufacturer’s protocol.

### Western Blotting

Total protein was extracted from frozen liver with RIPA lysate. The protein concentration was then detected by using the Pierce BCA Protein Assay Kit (23227, Thermo Scientific). Equal amount of protein extracts was separated by electrophoresis on a 9% SDS-PAGE gel, and proteins were then transferred onto nitrocellulose membranes and immunoblotted with primary antibodies at 4°C for overnight. Primary antibodies used were as follows: anti-METTL3 (ProteinTech, 15073-1-AP); anti-METTL14 (ABclonal, A8530); anti-FTO (ProteinTech, 27226-1-AP); anti-YTHDF3 (AVIVA, ARP55530); and anti-β actin (Bioworld, LM1713). The membranes were then immunoblotted with the second antibodies at room temperature for 2 h. Reactive signals were detected by Pierce ECL Western Blotting Substrate (32109, Thermo Scientific). ECL signals were visualized with an imaging system (Bio-Rad, USA) and normalized to β actin by using ImageJ software.

### N^6^-methyladenosine Immunoprecipitation and Library Construction

For genome-wide m^6^A profiling, approximately 400 µg of total RNA was extracted from liver using TRIzol in accordance with the manufacturer’s instructions. RNA quality was controlled by using Nano Drop ND-2000 and agarose gel electrophoresis. High-throughput m^6^A and mRNA sequencing were performed by LC Sciences, Inc. (Hangzhou, China) following the published procedure. Briefly, mRNA was firstly purified by using oligo (dT)-coupled magnetic beads. Following purification, the poly(A) mRNA fractions were then fragmented into ∼100-nt-long oligonucleotides using Magnesium RNA Fragmentation Module (NEB, cat. e6150, USA) under 86°C for 7 min. Then, equal amount of cleaved RNA fragments (in biological duplicates) from Control and LPS-treated group were subjected to m^6^A immunoprecipitation by incubating with anti-m^6^A antibody (No. 202003, Synaptic Systems, Germany) for 2 h at 4°C in IP buffer (50 mM Tris-HCl, 750 mM NaCl, and 0.5% IGEPAL CA-630). Rabbit normal IgG was used as the negative control. An equal amount of fragmented mRNA from each biological sample was reserved as the input control (Input). The eluted m^6^A-containing fragments (IP) and the input were converted by SuperScript^TM^ Ⅱ Reverse Transcriptase (Invitrogen, cat. 1896649, United States) to obtain cDNA, which was then used to synthesize U-labeled second-stranded DNAs with *E. coli* DNA polymerase Ⅰ (NEB, cat. m0209, United States), RNaseH (NEB, cat. m0297, United States) and dUTP solution (Thermo Fisher, cat. R0133, United States). A-bases were then added to the blunt ends of each strand for the subsequent ligation to the indexed adapters. Each adapter contains a T-base overhang for ligating the adapter to the A-tailed fragmented DNA. Single- or dual-index adapters were ligated to the fragments, and size selection was performed with AMPureXP beads. After the heat-labile UDG enzyme (NEB, cat. m0280, United States) treatment of the U-labeled second-stranded DNAs, the ligated products are amplified with PCR by the following conditions: initial denaturation at 95°C for 3 min; eight cycles of denaturation at 98°C for 15 s, annealing at 60°C for 15 s, and extension at 72°C for 30 s; and then final extension at 72°C for 5 min. The average insert size for the final cDNA library was 300 ± 50 base pairs (bp). At last, a 2 × 150-bp paired-end sequencing was performed on an illumina Novaseq™ 6,000 (LC-BioTechnology Co., Ltd., Hangzhou, China) following the vendor’s recommended protocol.

### Bioinformatics Analysis of m^6^A Sequencing and mRNA Sequencing Data

Fastp software was used to remove low-quality bases, undetermined bases, and reads containing adaptor contamination. Hisat2 software was applied to align reads to the reference genome of chicken. Mapped reads of IP and Input libraries were provided for R package exomePeak for m^6^A peak identified. MEME and HOMER were used for *de novo* and known motif finding, followed by localization of the motif with respect to peak summit. Called peaks were annotated by intersection with gene architecture by using R package ChIPseeker. Then, StringTie was applied to determine the expression level for all mRNAs from Input libraries by calculating FPKM. Differentially expressed mRNAs were selected with log2 (fold change) > 1 or log2 (fold change) < −1 and *p*-value < 0.05 by R package edge R.

### Statistical Analysis

Data were presented as the mean ± SE. Statistical analysis was done using SPSS 22.0 and GraphPad Prism 8.0 softwares, Paired Student’s t-tests were performed between LPS-treated and control samples. Difference with *p* < 0.05 was defined as the threshold for significance.

## Results

### LPS Treatment Altered the Overall m^6^A Level in RNA and the Expression of m^6^A Associated Factors

**FIGURE 1:**
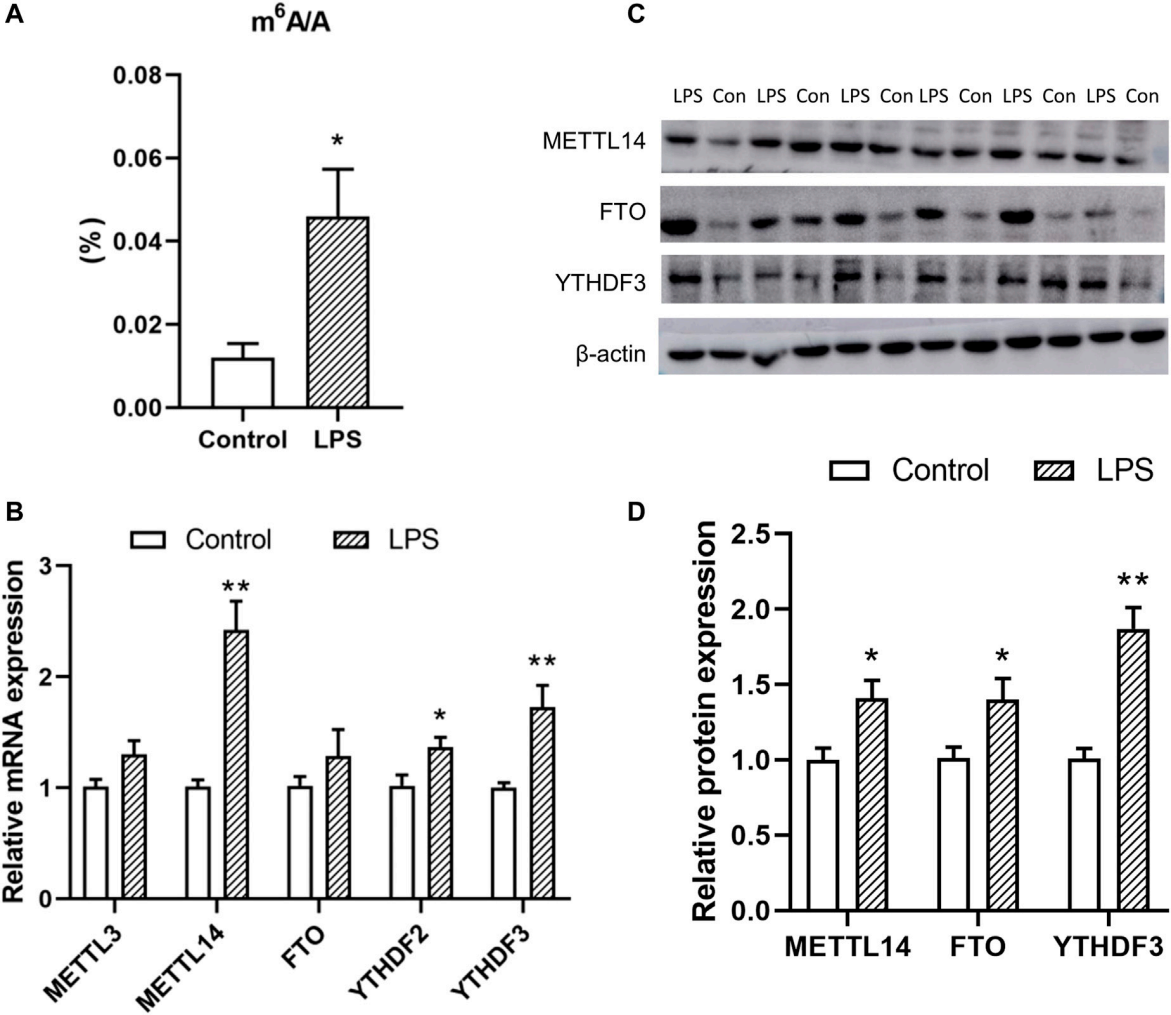
Effects of LPS on the total m^6^A level **(A)** and the expression of the associated enzymes [**(B)** mRNA and **(C,D)** protein level]. Values are mean ± SEM, *n* = 6/group, *p < 0.05, **p < 0.01.

The plasma triglycerides’ level 2 h after LPS treatment was increased compared to the control group (Supplementary Table S2), which indicated a rapid effect of LPS-induced inflammation on the lipid metabolism. Thus, the following tests were carried out on samples obtained 2 h after LPS treatment. Antibody-mediated capture of m^6^A and subsequent colorimetric analysis revealed that global m^6^A modification level in chicken liver was increased 2 h after the LPS treatment (Figure 1A). The expressions of m^6^A associated factors were then detected. The expression of the m^6^A writer METTL14 (*p* < 0.05) and reader YTHDF3 (*p* < 0.01) increased at the mRNA and protein levels after LPS treatment. Meanwhile, expression of FTO was increased at the protein level (*p* < 0.05) but not at the mRNA level.

### Transcriptome-wide m^6^A-Seq Revealed Global m^6^A Modification Patterns in Chicken Liver

**FIGURE 2:**
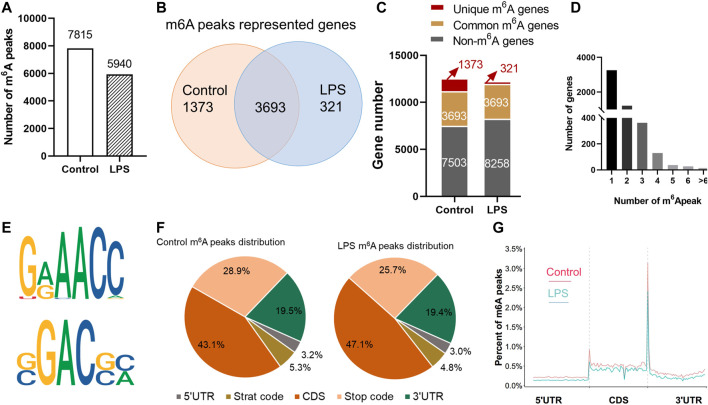
Transcriptome-wide m6^A^-Seq of m^6^A peaks in chicken liver. **(A)** Number of total m^6^A peaks identified in control and LPS group; **(B,C)** m^6^A modified genes in control and LPS group. **(D)** Number of m^6^A peaks in each modified gene. **(E)** The top consensus motif of the control m^6^A peaks. **(F)** The ratio of m^6^A distribution in different region of the transcripts both in control and LPS group. **(G)** Distribution of m^6^A peaks across the length of mRNA transcripts. Each transcript is divided into three parts: 5′UTRs, CDs, and 3′UTRs.

MeRIP-Seq identified 7,815 m^6^A peaks representing 5,066 genes of chicken liver in the control group. In the LPS-treated group, 5,940 peaks were identified and represented the transcripts of 4,014 genes (Supplementary Table S3). According to the differences and overlaps in m^6^A modified genes, 321 genes were uniquely modified in the LPS-treated group, and 3,693 of them were commonly distributed in both groups. The maximum m^6^A number in all transcripts was 11, and the majority of genes (4,499/5,066) had one or two m^6^A modified sites (Figure 2D). The top 15 genes that contain the most m^6^A peaks were shown in Supplementary Table S4.

The m^6^A methylomes were further mapped by HOMER software. The top consensus motifs in the 7,815 peaks in the control group were AAACC and GGACG (Figure 2E). The distribution of the m^6^A peaks in the transcriptome of control and LPS group was also analyzed. Transcripts were divided into 5′UTR, start code, CDS, stop codon, and 3′UTR. m6A peak distribution showed similar patterns in control and LPS groups. In general, m6A peaks were highly distributed in the CDS and 3′UTR and less abundant in 5′UTR (Figure 2F). The m^6^A peak was most correlated with two regions: in the vicinity of stop codon and the start code region. Approximately 20% of CDS m^6^A peaks were distributed in the first exon, and the percentage of m^6^A peaks was stable along the transcript length but increases dramatically at the end of the CDS at threefold higher than at the beginning. In the 3′UTR, the peaks were enriched near the stop codon, then dramatically decreased, and maintained a low level along the length of 3′UTR (Figure 2G).

### Abnormal m^6^A-Modified Genes Were Enriched in Immune and Metabolic Process

**FIGURE 3:**
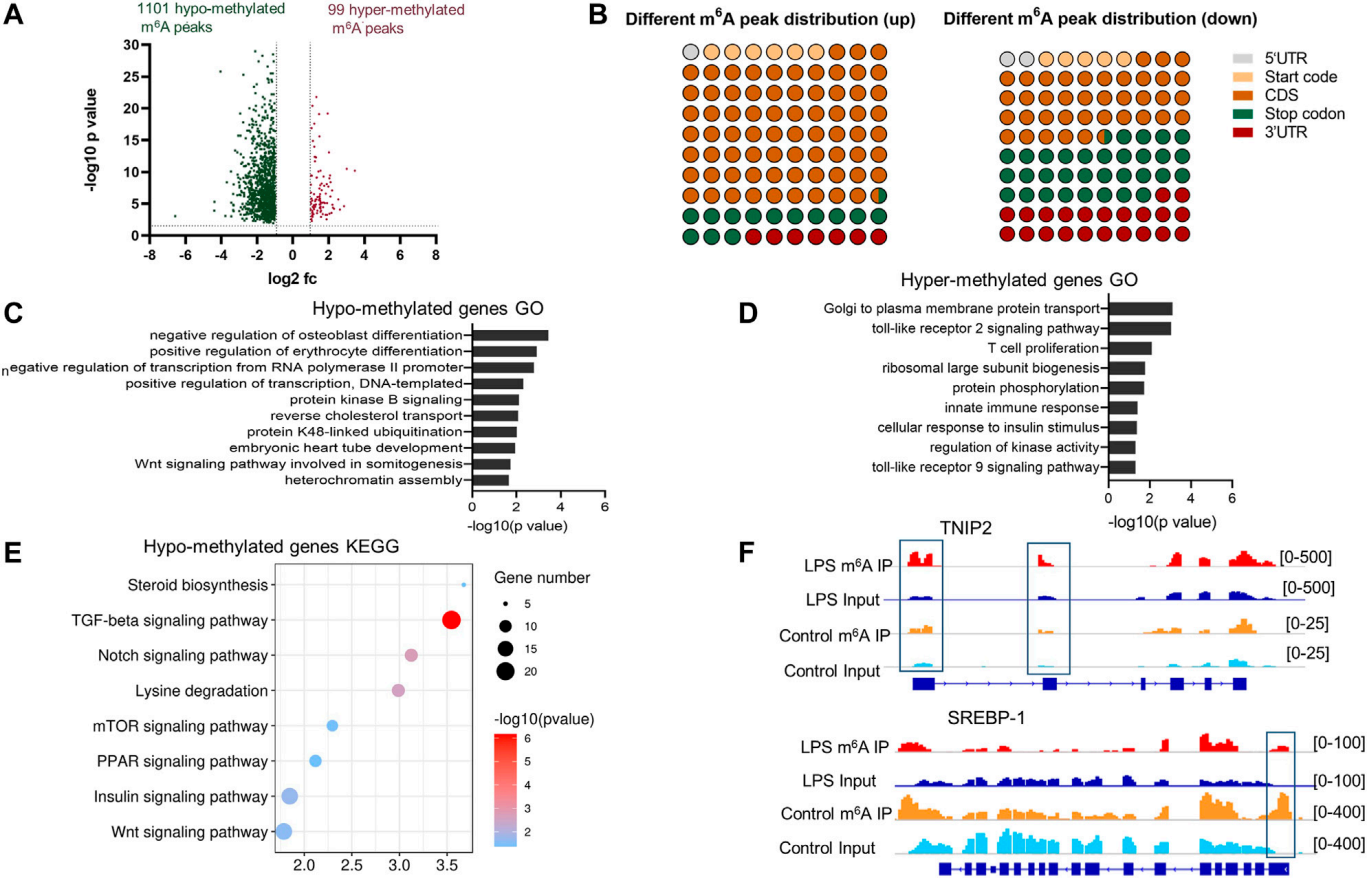
Global m^6^A modification changes in liver of LPS-treated group compared with normal group. **(A)** Hyper-methylated and hypo-methylated m^6^A peaks identified in the LPS group (fold change > 2, *p* < 0.05). **(B)** The distribution of the differentially methylated peaks along the transcripts. **(C,D)** Gene ontology analysis of biological process of the transcripts with hypo-methylated m^6^A peaks and hyper-methylated m^6^A peaks, respectively. The enriched GO terms were shown in columns with −log10 (*p*-value) indicating the significance. **(E)** Gene set enrichment pathway analysis of transcripts with hypo-methylated m^6^A peaks in LPS-treated samples. **(F)** The m^6^A abundances in TNIP2 and SREBP-1 mRNA transcripts in Control and LPS samples, as detected by m^6^A-seq. The blue rectangles indicated the m^6^A peaks that have a significant increased or decreased abundance (fold change>2, *p* < 0.05) in LPS samples comparing with the Control samples.

The comparison of the abundance of the m^6^A peaks between LPS group and control revealed 1,200 differentially methylated m^6^A peaks distributed in 1,039 genes (fold change > 2, *p* < 0.05) (Supplementary Table S3). A total of 1,101 hypo- and 99 hyper-methylated m^6^A peaks were found in the LPS group (Figure 3A). Analysis of abnormal m^6^A peaks distribution showed that the hyper-methylated sites were mostly in the CDS region, and the hypo-methylated sites preferentially in the region nearing the stop codon and the 3′UTR (Figure 3B).

GO and KEGG analyses of hypo- and hyper-methylated peaks were conducted using the DAVID 6.8 database (http://david.ncifcrf.gov/), and the results were considered statistically significant when *p* < 0.05. For hypo- and hyper-methylated genes, 32 and 9 significantly enriched GO items were identified, respectively. The top 10 hypo-methylated gene oncology items and all the hyper-methylated gene oncology items are shown in Figures 3C,D, respectively. The hyper-methylated genes were mostly enriched in the immune reaction-associated items, such as TLR2 signaling pathway, T cell proliferation, innate immunity, and TLR9 signaling pathway. The hypo-methylated genes were enriched in the items related to cell development and gene transcription. KEGG analysis of the hypo-methylated genes revealed eight pathways that were significantly enriched, namely, steroid biosynthesis, TGFβ signaling pathway, Notch signaling pathway, lysine degradation, mTOR signaling pathway, PPAR signaling pathway, insulin signaling pathway, and Wnt signaling pathway (Figure 3E). No significantly enriched pathway was identified when analyzing the hyper-methylated genes possibly due to the small number of genes.

### Identification of Differentially Expressed Genes in LPS-Treated Group

**FIGURE 4:**
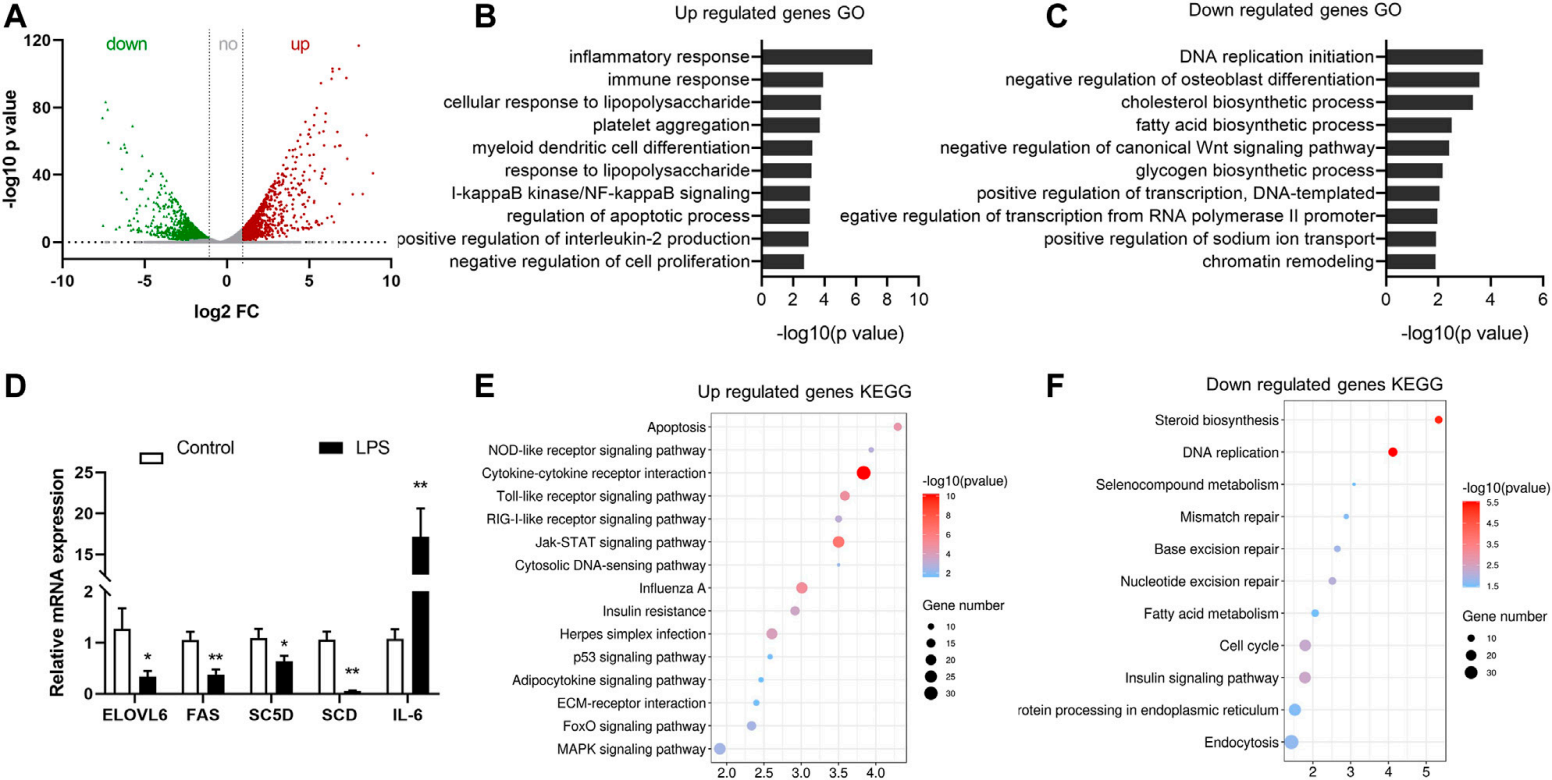
Identification of differentially expressed genes in LPS-treated group by RNA-seq. **(A)** Upregulated and downregulated genes in the LPS group compared with the control (fold change > 2, *p* < 0.05). **(B,C)** Gene ontology analysis of biological process of the differentially expressed genes. The top 10 enriched GO terms were shown in columns with −log10 (*p*-value) indicating the significance. **(D)** Real-time PCR validation of the expression of genes. **(E,F)** Gene set enrichment pathway analysis of genes differently expressed in LPS-treated samples.

In the RNA sequencing dataset (m^6^A-Seq Input library), 2,854 differently expressed genes were detected during the acute liver inflammation induced by LPS. Among them, 917 genes were upregulated and 1,948 genes were downregulated (fold change > 2, *p* < 0.05). Data scatter plot o was shown in Figure 4A. Gene ontology and pathway analysis revealed that the upregulated genes in the LPS-treated group were significantly enriched in biological process and pathways closely related to immune response (Figures 4B,E). This finding was consistent with the acute phase response of chicken under LPS stimulation. The downregulated genes were mostly enriched in metabolic process and DNA replication and transcription (Figures 4C,F). Moreover, the lipid metabolism-associated process was specially enriched, such as cholesterol biosynthetic process and fatty acid biosynthetic process. Real-time PCR validated the expression of several genes, and the results agreed with the sequencing data.

### Conjoint Analysis of m^6^A-RIP-Seq and RNA-Seq Data of the LPS-Treated Samples and Control

**FIGURE 5:**
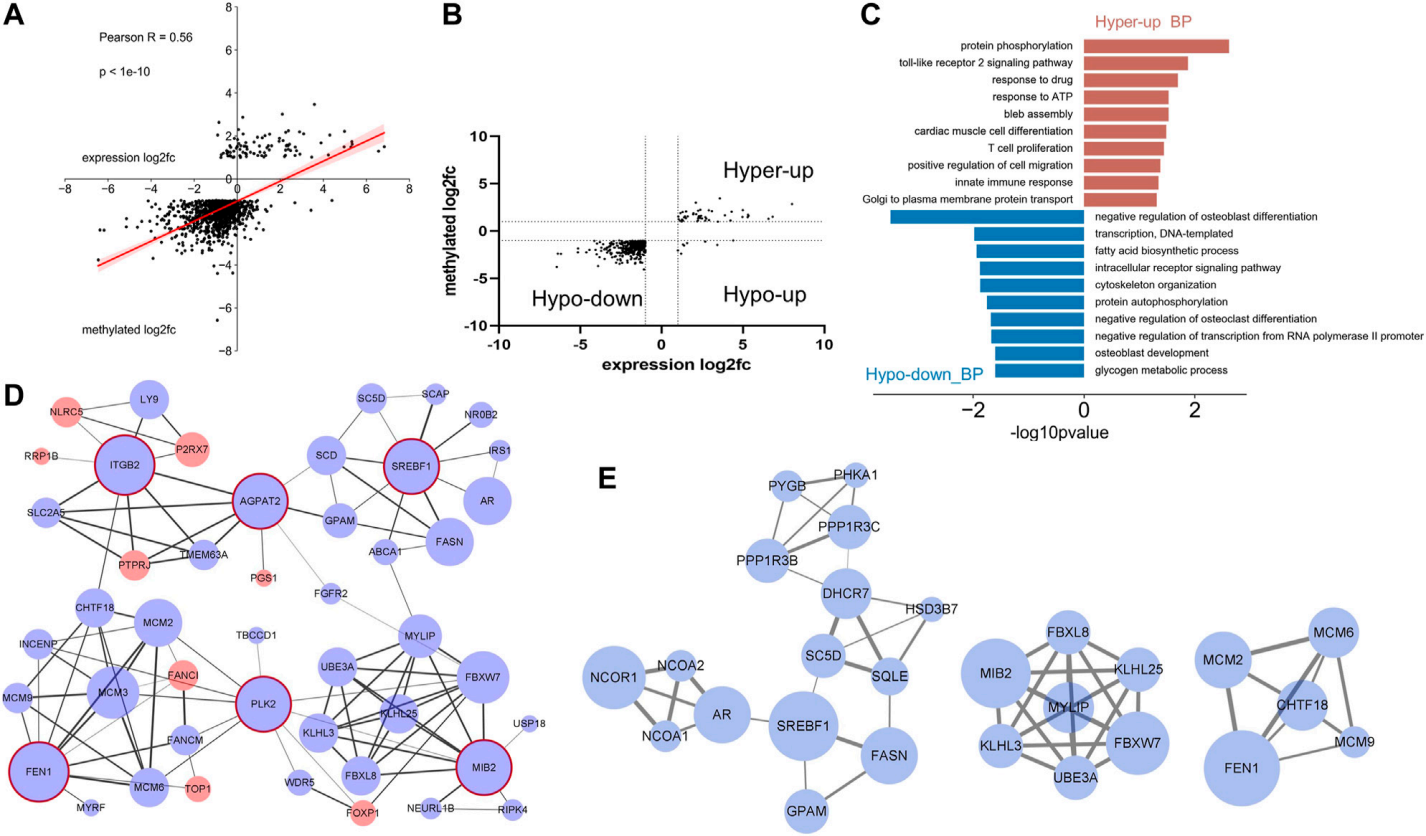
Conjoint analysis of m6A-RIP-seq and RNA-seq data of the LPS-treated samples and control. **(A)** Dot plot of log2 fold change of mRNA expression against log2 fold change of differential m^6^A methylation. **(A)** Positive correlation was shown (Pearson r = 0.56). **(B)** Distribution of genes with significant changes in both m^6^A and mRNA levels in LPS-treated group compared with adjacent normal group (fold change > 2, *p* < 0.05). **(C)** Gene ontology analysis of biological process of the differentially expressed and m^6^A methylated genes. **(D)** Graph of the protein–protein interaction network of the genes [mentioned in **(B)**] conducted by Cytoscape software. Red and blue circles represent the up- and down-expressed genes, respectively. The circles with red edge line were hub genes. Different width values of the lines between two nodes indicated different combined scores, and the wider line represents higher score. **(E)** Modules of the PPI network.

Correlation between the differentially methylated genes and the corresponding mRNA expression levels was analyzed by combining m^6^A-seq and RNA data. The m^6^A methylation level was significantly positively correlated with the gene expression level (Figure 5A). As most of the differently regulated peaks distributed in the CDS, STOP, and 3′UTR region, we analyzed the possible relations between different peaks in these regions and the corresponding mRNA expressions. The results showed that the methylation level in these regions was also positively related to the mRNA expression (Supplementary Figure S1). A total of 406 genes were differentially methylated and differentially expressed (*p* < 0.05, fold change >2). Most of these genes were hypo-methylated and downregulated (351/406), and many were hyper-methylated and up-expressed (47/406). Not one gene was hyper-methylated and down-expressed. Only eight genes showed hypo-methylation and up-expression (Figure 5B).

The 398 hypo-down or hyper-up genes were subjected to GO analysis to find their related biological process. As shown in Figure 5C, the hyper-up transcripts were involved in several immunity-related processes. This finding is consistent with the result in Figure 3D, showing that the hyper-methylated genes were correlated with the immune response under LPS treatment. Meanwhile, significant differences in enriched biological processes were observed between hypo-down and hypo-methylated gene (Figure 3C). Here, the process related osteoblast and osteoclast were remarkably enriched. Fatty acid biosynthetic process was also enriched (Figure 5C).

The prediction of protein–protein interaction (PPI) of the differentially methylated and expressed genes was carried out by using the STRING online database, and the results were downloaded and analyzed using Cytoscape software. The following top six hub genes (degree ≥ 10) and their corresponding first-stage node were screened: FEN1, PLK2, MIB2, AGPAT2, SREBF1, and ITGB2. The network was clustered into four subparts around the hub genes. Genes interacting with the hub gene ITGB2 (also called CD18), such as NLRC5, LY9, P2RX7, and PTRRJ, were mostly related to immune response. Genes interacting with SREBF1 were closely involved in the lipid metabolism. The other two subparts were largely associated with protein ubiquitination and DNA replication, respectively (Figure 5D). By using MCODE, several functional modules were screened, and modules enriched in lipid metabolism, DNA replication, and protein ubiquitination were re-emerged (Figure 5E). These results indicated that the m^6^A modification may participate in these biological processes in the acute stage after LPS stimulation.

## Discussion

Liver is one of the most important metabolic organs and is also a frontline immune tissue. A close association between inflammation and abnormal lipometabolism has been widely confirmed, and various signal pathways and genes have been revealed to uncover the mechanisms underlying the inflammation-induced abnormal lipometabolism. Here, a novel path considering the role of m^6^A modification was explored. With the use of ELASE-based kit, the global m^6^A level of hepatic RNA was found to be significantly induced 2 h after LPS treatment. Similar results that LPS induced the global m^6^A level have been previously reported in pig liver, human dental pulp cells, and microglia cells (Feng et al., 2018; Lu et al., 2018; Wen et al., 2020). However, varying results had been published regarding the expression levels of responsible m^6^A enzymes. The protein expression of METTL14, FTO, and YTHDF3 was increased in this study. Previous studies showed that LPS treatment induced the expression of METTL3 mRNA and protein in human dental pulp cells (Feng et al., 2018) and microglia cells (Wen et al., 2020), and LPS-treated osteoblast showed a decreased METTL3 mRNA and protein expression (Zhang et al., 2019). LPS treatment for macrophages induced the expression of YTHDF2 (Wang et al., 2019). This diversity of responsible m^6^A enzymes after LPS stimulation suggested varying mechanisms of m^6^A in the LPS-induced inflammation in different cell lines or species. All these findings revealed that m^6^A and the associated modulators play important role in the LPS-induced inflammation response in different cell types and species.

m^6^A modification also plays important roles in the lipid metabolism regulation. Hu et al. (2010) found that m^6^A and the eraser FTO participate in the GR-induced hepatic lipid accumulation in chicken (Hu et al., 2020). In piglets, the dietary supplementation of branched-chain amino acids decreases lipid accumulation, accompanied with the increase in m^6^A level and protein expression of METTL3 and METTL14 in liver (Heng et al., 2020). Considering that m^6^A is closely associated with immune response and lipid metabolism, we hypothesized that m^6^A may function as a link in chicken liver, mediating the inflammation-induced abnormal lipid metabolism. Here, altered lipid metabolism during LPS intraperitoneal treatment was confirmed by the wide difference in the expression of lipid metabolism-associated genes as revealed by RNA-seq. GO and KEGG analysis also identified the significantly enriched pathways associated with immune response or lipid metabolism.

m^6^A modification pattern in chicken liver was then identified at the normal and inflammation state. A total number of 7,815 m^6^A peaks distributed in 5,066 gene transcripts were identified in chicken liver under normal condition. In a previous study, 12,769 m^6^A peaks within 6,990 gene transcripts have been identified in HepG2 cells (Dominissini et al., 2012). The number of m^6^A peak and the m^6^A-containing transcripts in chicken liver were both low. However, the comparison of the average m^6^A peak number of the m^6^A modified genes revealed similarity in the previous and present findings, that is, ∼1.5 in chicken and ∼1.7 in HepG2 cells. Most of the m^6^A-modified genes possess one or two peaks that are mostly tended to be located in the region of CDS and 3′UTR, especially around the start codon and the stop codon. This distribution characteristic and the RRACH conserved sequence motif feature were consistent with previous studies (Luo et al., 2014; Chen et al., 2020; Hu et al., 2020), thus suggesting a high m^6^A modification conservation among different species.

The m^6^A modification pattern in the liver of LPS-treated chicken differed from that in the normal controls. A total number of 1,200 different m^6^A peaks were found, of which many m^6^A sites were hypo-methylated and preferred to occur in the 3′UTR and near the stop codon regions. Analysis on the differently methylated transcripts revealed that the hyper-methylated genes were significantly enriched in the immune-associated biological processes, and the hypo-methylated genes were involved in many pathways related with metabolism such as steroid biosynthesis, TGFβ signaling pathway, mTOR signaling pathway, PPAR signaling pathway, and insulin signaling pathway. The global change of m^6^A modification profiles could deal to the abnormal expression of m^6^A enzymes, as we found in this study that the protein expression of METTL14, FTO protein, was significantly changed in chicken liver after LPS stimulation.

Although the m^6^A modification pattern was changed and the differentially methylated transcripts were enriched in inflammation and metabolism process, whether m^6^A mediates the inflammation-induced lipid metabolism disorder remains unclear. Therefore, a combined analysis of m^6^A-seq and mRNA-seq data was conducted. Correlation analysis confirmed a significantly positive relationship between m^6^A modification and the mRNA expression. This observation was consistent with some previous studies (Luo et al., 2014; Chen et al., 2020) but different from others (Schwartz et al., 2014; Wang et al., 2014). It may be due to the differences in species and sample collection. Note that the differentially m^6^A modified genes were mostly hypo-modified, and the hypo-m^6^A peaks tended to occur in the 3′UTR and in the region nearing the stop codon (Figures 3A,B). It has been revealed that m^6^A affects RNA bioprocesses, such as RNA splicing, export, translation, and stability. 3′UTR is well known to regulate mRNA stability, localization, and translation (Mayr, 2019). The higher frequency of different m^6^A peak localized in the 3′UTR region suggested the important role of m^6^A in the function of 3′UTR.

The putative PPI for the 406 transcripts with different m^6^A modification and mRNA expression was predicted. The genes associated with immune response, lipid metabolism, protein ubiquitination, and DNA replication were clearly clustered. ITGB2 was the hub gene associated with immune response. This gene codes the protein widely expressed in immune cells such as dendritic cells, monocytes, macrophages, neutrophiles and B cells, and activates proinflammatory pathways, resulting in production of inflammatory cytokines (Davalos and Akassoglou, 2012). SREBF1 was the hub gene associated with lipid metabolism. The protein encoded by this gene is deeply involved in the lipid metabolism regulation (Khesht and Hassanabadi, 2012). According to the PPI network (Figure 5D), the genes connected with SREBP-1 including SCD, SC5D, FAS, and GPAM are all important enzymes involved in lipid metabolism. Hu et al. showed that subcutaneous corticosterone injection in chicken decreases the mRNA expression and m^6^A modification level of SREBP-1, FAS, and SCD (Hu et al., 2020). These results further suggested the connecting role of m^6^A in inflammation-induced lipid abnormality, and the SREBP-1 might be a target in the regulatory process of m^6^A.

AGPAT2 is a hub gene that seems to link the inflammation cluster with the lipid metabolism cluster. This gene encodes an enzyme whose deficiency will cause congenital generalized lipodystrophy characterized by severe lipoatrophy, insulin resistance and hepatic steatosis (Garg and Agarwal, 2009). Tapia et al. reported that the AGPAT2 absence in mice brown adipocytes decreases lipid load and the expression of PPARγ, PPARα, C/EBPα, and PGC1α, key regulators of lipid metabolism. A significantly increased mRNA abundance was also found for ISGs, which participate in IFN type Ⅰ response (Tapia et al., 2020). Although the mechanism of the increased levels of ISGs in AGPAT2 deficient brown adipocytes remains unknown, it is easy to raise the hypothesis that AGPAT2 plays a role in connecting the lipid metabolism with immune response. The present results may provide additional support to this hypothesis. As the PPI was limitedly predicted by computational approach, further analyses including the validation of the m^6^A modification level by using MeRIP-qPCR are need to clarify the role of SREBP-1 and AGPAT2 in the process of inflammation-induced lipid metabolism alteration.

In this study, the wide range variation of m^6^A modification and gene expression were observed at 2 h after LPS treatment, and this finding is consistent with the feature of post-translational regulation of gene expression. Until today, adverse environmental exposures such as particulate matter, cigarette smoke, UV, metal-induced inflammation, and reactive oxygen species were shown to mediate m^6^A modification, thereby regulating downstream gene expression and signaling pathways (Li et al., 2020). These results indicated a role of m^6^A in the acute stage response of animals and plants amid the environmental stress. Although the molecular mechanisms on how m^6^A regulates hub genes expression in different conditions remain to be elucidated, m^6^A has potential applications as a target for exploring the mechanism inflammation-induced abnormal lipid metabolism and may also serve as a disease biomarker.

## Conclusion

This study presented the m^6^A transcriptome-wide map of broiler liver under normal condition and LPS-induced acute inflammatory stage and suggested a potential mechanism of LPS-induced acute inflammation and the linked abnormal lipid metabolism.

## Data Availability

The original contributions presented in the study are publicly available. These data can be found here: GSE189591.

## Abbreviations

AGPAT2, 1-acylglycerol-3-phosphate O-acyltransferase 2; AGPAT2, 1-acylglycerol-3-phosphate O-acyltransferase 2; FAS, fatty acid synthase; FEN1, flap structure-specific endonuclease 1; FTO, fat mass and obesity associated gene; GPAM, glycerol-3-phosphate acyltransferase. ITGB2, integrin subunit beta 2; LPS, lipopolysaccharide; LY9, lymphocyte antigen 9; METTL3, methyltransferase-like 3; MIB2, MIB E3 ubiquitin protein ligase 2; NLRC5, NLR family CARD domain containing 5; P2RX7, purinergic receptor P2X 7; PLK2, polo-like kinase 2; PTRRJ, protein tyrosine phosphatase receptor type J; SC5D, sterol-C5-desaturase; SCD, stearoyl-CoA desaturase; SREBF1, sterol regulatory element binding transcription factor 1; YTHDF2, YTH domain family 2.
