# Supplementary material for: m6A mRNA Methylation Was Associated With Gene Expression and Lipid Metabolism in Liver of Broilers Under Lipopolysaccharide Stimulation
Source: Front Genet. 2022 Feb 25;13:818357. doi: 10.3389/fgene.2022.818357 (PMC8914017; doi:10.3389/fgene.2022.818357)
Supplement: Supplementary file 2 [file Table1.docx]

**Supplementary table 1 Nucleotide sequences of primers used in the Real-Time PCR**

| Target gene | Sequence (F: forward, R:reverse, 5’-3’) |
| --- | --- |
| METTL3 | F: GAGATCTACGGGATGATCG R: AATTGGTTGCCTAAAGTGA |
| METTL14 | F: GTGGTTCAGGAGAGGGTC R: TCGTTTTTCCAGGATTAT |
| FTO | F: TGAAGGTAGCGTGGGACATAGA R: GGTGAAAAGCCAGCCAGAAC |
| YTHDF2 | F: TCCTACTCTCTGGGTGAGGC R: GCGTAATTGCTGCTGTAGCC |
| YTHDF3 | F: TACTCTTTAGGCGAAGCGGC R: GAAACGGAGGGGTGTTTCCT |
| ELOVL6 | F:GACCACTAAAGAAGACGCT R:GCTGACAATTCACAGAACG |
| FAS | F: TGAAGGACCTTATCGCATTGC R: GCATGGGAAGCATTTTGTTGT |
| SC5D | F:CTTCTGGACACACTGGGACT R:TTGGCAAATGAGCTTTATTA |
| SCD | F: CTTCATTCCAGCCATCCTGC R: CGCTCTTGTGACTCCCATCT |
| IL-6 | F: GAAATCCCTCCTCGCCAATCTG R:GCCCTCACGGTCTTCTCCATAAA |
