# Supplementary material for: m6A mRNA Methylation Was Associated With Gene Expression and Lipid Metabolism in Liver of Broilers Under Lipopolysaccharide Stimulation
Source: Front Genet. 2022 Feb 25;13:818357. doi: 10.3389/fgene.2022.818357 (PMC8914017; doi:10.3389/fgene.2022.818357)
Supplement: Supplementary file 3 [file Table2.docx]

**Supplementary** **table 2 The plasma and hepatic Tg and Tch level**

| Parameters |  | 2 h | | 24 h | |
| --- | --- | --- | --- | --- | --- |
|  |  | Control | LPS | Control | LPS |
| Liver | Tg (mmol/g) | 20.70 ± 2.14 | 18.99 ± 1.58 | 11.87 ± 0.71 | 14.92 ± 1.48* |
|  | Tch (mmol/g) | 4.21 ± 0.10 | 4.48 ± 0.21 | 5.07 ± 0.50 | 5.31 ± 0.56 |
| Plasma | Tg (mmol/L) | 0.48 ± 0.03 | 0.54 ± 0.04 (*P* = 0.09) | 0.64 ± 0.04 | 0.73 ± 0.08 |
|  | Tch (mmol/L) | 7.66 ± 0.26 | 7.00 ± 0.33 | 5.89 ± 0.25 | 6.57 ± 0.28 (*P* = 0.09) |

* means significant difference between LPS treated group and the control group at the same time point
