# Supplementary material for: m6A mRNA Methylation Was Associated With Gene Expression and Lipid Metabolism in Liver of Broilers Under Lipopolysaccharide Stimulation
Source: Front Genet. 2022 Feb 25;13:818357. doi: 10.3389/fgene.2022.818357 (PMC8914017; doi:10.3389/fgene.2022.818357)
Supplement: Supplementary file 4 [file Table4.docx]

**Supplementary** **table 4 The top 13 genes that contain** **the most m^6^A peaks**

| Gene symbol | Full name of the gene | Peak number |
| --- | --- | --- |
| BAHCC1 | BAH domain and coiled-coil containing 1 | 11 |
| TNRC18 | trinucleotide repeat containing 18 | 11 |
| NES | nestin | 9 |
| ANKRD11 | ankyrin repeat domain 11 | 9 |
| ZC3H13 | zinc finger CCCH-type containing 13 | 8 |
| SETD2 | SET domain containing 2 | 8 |
| TCOF1 | treacle ribosome biogenesis factor 1 syndrome 1 | 7 |
| C4 | complement 4 precursor | 7 |
| FTSJ3 | FtsJ RNA 2'-O-methyltransferase 32'-O-methyltransferase 3 | 7 |
| NCOR2 | nuclear receptor corepressor 2 | 7 |
| MYH10 | Myosin heavy chain 10 | 7 |
| MED1 | mediator complex subunit 1 | 7 |
| GSE1 | Gse1 coiled-coil protein | 7 |
