# Supplementary figures and images for: m6A mRNA Methylation Was Associated With Gene Expression and Lipid Metabolism in Liver of Broilers Under Lipopolysaccharide Stimulation
Source: Front Genet. 2022 Feb 25;13:818357. doi: 10.3389/fgene.2022.818357 (PMC8914017; doi:10.3389/fgene.2022.818357)

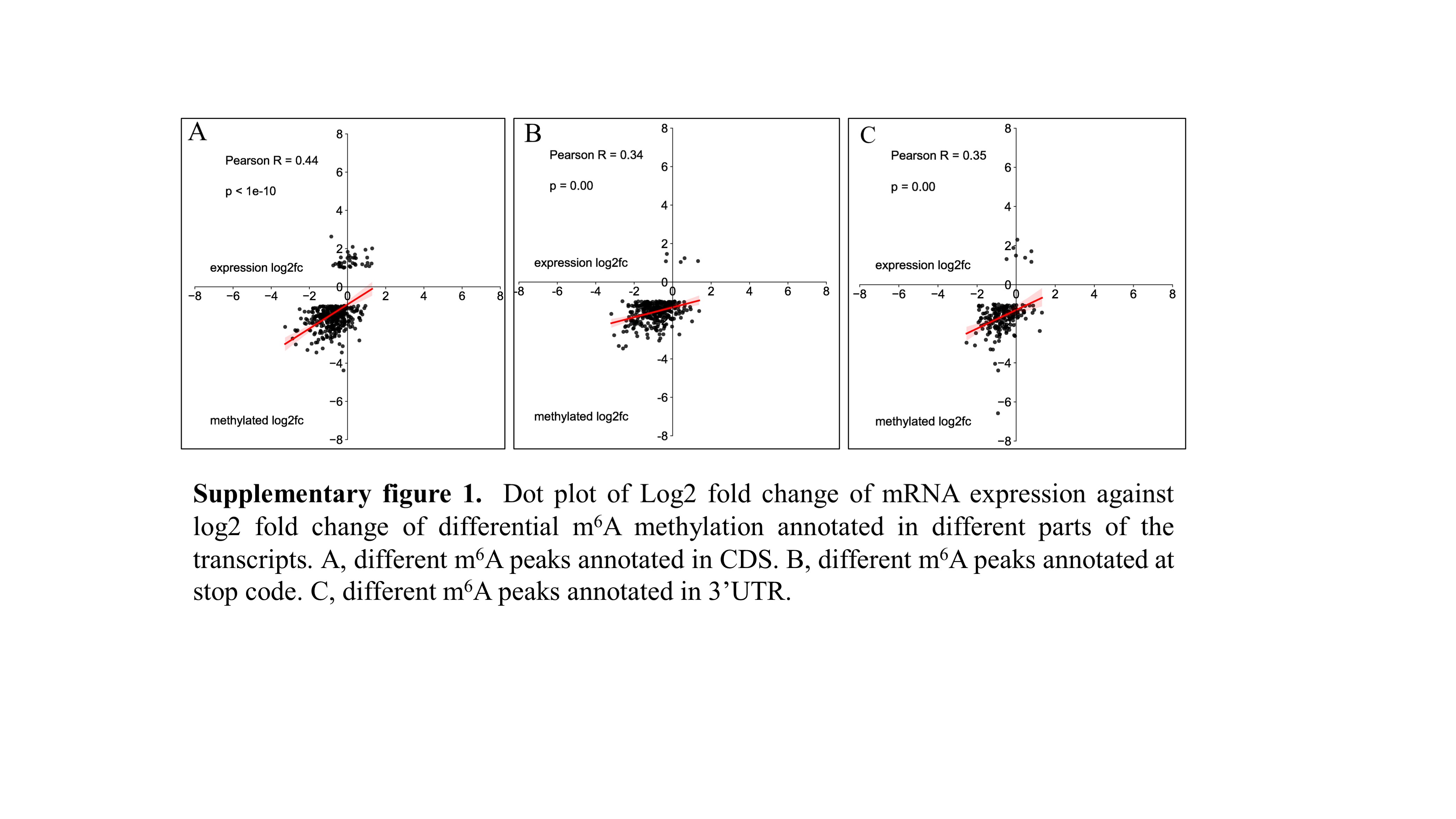

Supplement: Supplementary file 5 [file Image1.jpg]
